# Poly[[hexa­aqua­(μ_2_-oxalato-κ^4^ *O* ^1^,*O* ^2^:*O* ^1′^,*O* ^2′^)bis(μ_3_-pyridine-2,4-dicarboxyl­ato-κ^4^ *N*,*O* ^1^:*O* ^1′^:*O* ^4^)dicerium(III)] monohydrate]

**DOI:** 10.1107/S1600536811051956

**Published:** 2011-12-07

**Authors:** Fwu Ming Shen, Shie Fu Lush

**Affiliations:** a Department of Biotechnology, Yuanpei University, HsinChu 30015, Taiwan; b Department of General Education Center, Yuanpei University, HsinChu 30015, Taiwan

## Abstract

In the polymeric title compound, {[Ce_2_(C_7_H_3_NO_4_)_2_(C_2_O_4_)(H_2_O)_6_]·H_2_O}_*n*_, the Ce^3+^ cation is nine-coordinated in a distorted CeNO_8_ tricapped trigonal–prismatic geometry, formed by three pyridine-2,4-dicarboxyl­ate anions, one oxalate anion and three water mol­ecules. The mid-point of the oxalate anion is located on an inversion center. The oxalate and pyridine-2,4-dicarboxyl­ate anions bridge the Ce^3+^ cations, forming a two-dimensional polymeric complex parallel to (010). Inter­molecular classical O—H⋯O hydrogen bonding and weak C—H⋯O hydrogen bonding are present in the crystal structure and π–π stacking [centroid–centroid distance = 3.558 (2) Å] is observed between parallel pyridine rings of adjacent mol­ecules. The uncoordinated water mol­ecule shows an occupancy of 0.5.

## Related literature

For the isotypic La^3+^ complex, see: Shen & Lush (2011 ▶). For related pyridine-2,4-dicarboxyl­ate complexes, see: Aghabozorg *et al.* (2011 ▶); Li *et al.* (2007 ▶); Wang *et al.* (2009 ▶).
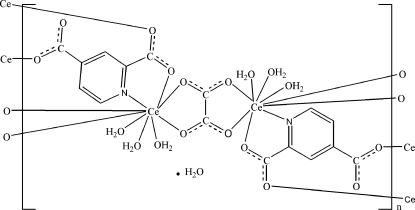


## Experimental

### 

#### Crystal data

[Ce_2_(C_7_H_3_NO_4_)_2_(C_2_O_4_)(H_2_O)_6_]·H_2_O
*M* *_r_* = 824.58
Triclinic, 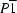

*a* = 6.4160 (5) Å
*b* = 6.6486 (6) Å
*c* = 13.9920 (12) Å
α = 89.917 (1)°
β = 85.588 (1)°
γ = 73.676 (1)°
*V* = 570.98 (8) Å^3^
*Z* = 1
Mo *K*α radiation
μ = 4.04 mm^−1^
*T* = 294 K
0.30 × 0.10 × 0.10 mm

#### Data collection

Bruker SMART 1000 CCD area-detector diffractometer
Absorption correction: multi-scan (*SADABS*; Bruker, 2001 ▶) *T*_min_ = 0.639, *T*_max_ = 0.937
6072 measured reflections
2664 independent reflections
2416 reflections with *I* > 2σ(*I*)
*R*_int_ = 0.030

#### Refinement

*R*[*F* ^2^ > 2σ(*F* ^2^)] = 0.028
*wR*(*F* ^2^) = 0.081
*S* = 1.10
2664 reflections
177 parameters
H-atom parameters constrained
Δρ_max_ = 2.75 e Å^−3^
Δρ_min_ = −2.70 e Å^−3^

### 

Data collection: *SMART* (Bruker, 2007 ▶); cell refinement: *SAINT* (Bruker, 2007 ▶); data reduction: *SAINT*; program(s) used to solve structure: *SHELXTL* (Sheldrick, 2008 ▶); program(s) used to refine structure: *SHELXTL*; molecular graphics: *PLATON* (Spek, 2009 ▶); software used to prepare material for publication: *PLATON*.

## Supplementary Material

Crystal structure: contains datablock(s) global, I. DOI: 10.1107/S1600536811051956/xu5400sup1.cif

Structure factors: contains datablock(s) I. DOI: 10.1107/S1600536811051956/xu5400Isup2.hkl

Additional supplementary materials:  crystallographic information; 3D view; checkCIF report

## Figures and Tables

**Table 1:** Selected bond lengths (Å)

Ce—N1	2.684 (3)
Ce—O1^i^	2.409 (4)
Ce—O3^ii^	2.505 (3)
Ce—O4	2.511 (3)
Ce—O5	2.508 (3)
Ce—O6^iii^	2.515 (3)
Ce—O7	2.568 (5)
Ce—O8	2.515 (4)
Ce—O9	2.582 (5)

Symmetry codes: (i) 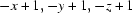; (ii) 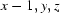; (iii) 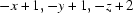.

**Table 2:** Hydrogen-bond geometry (Å, °)

*D*—H⋯*A*	*D*—H	H⋯*A*	*D*⋯*A*	*D*—H⋯*A*
O7—H7*A*⋯O4^ii^	0.86	2.03	2.879 (6)	171
O7—H7*B*⋯O10^iv^	0.83	1.84	2.569 (10)	146
O8—H8*A*⋯O6^v^	0.92	2.00	2.910 (5)	170
O8—H8*B*⋯O2^vi^	0.86	1.84	2.655 (6)	159
O9—H9*A*⋯O6^v^	0.99	2.01	2.987 (6)	169
O9—H9*B*⋯O10	0.84	1.93	2.440 (10)	118
O10—H10*A*⋯O5^ii^	0.84	2.12	2.844 (9)	143
O10—H10*A*⋯O8^ii^	0.84	2.39	2.913 (10)	121
O10—H10*B*⋯O9^vii^	0.94	1.63	2.501 (11)	153
C5—H5*A*⋯O3^ii^	0.93	2.46	3.131 (5)	129

Symmetry codes: (ii) 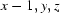; (iv) 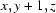; (v) 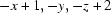; (vi) 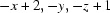; (vii) 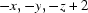.

**Fig. 1.**
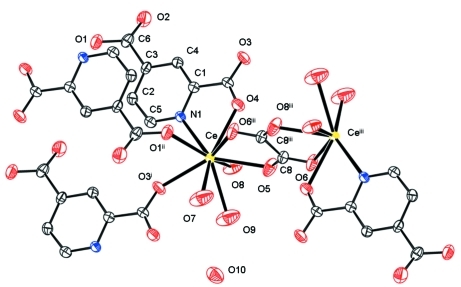
View of the title compound with the atom numbering scheme. Displacement ellipsoids for non-H atoms are drawn at the 50% probability level. All H atoms have been omitted for clarity. [Symmetry code: (i) x - 1, y, z; (ii) -x + 1, -y + 1, -z + 1; (iii) -x + 1, -y + 1, -z + 2.]

**Fig. 2.**
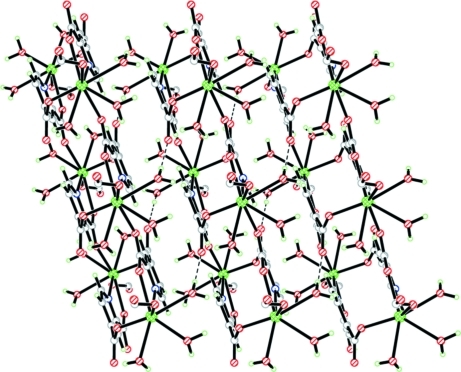
The crystal packing of (I) viewed along the c axis. Hydrogen bonds are shown as dashed lines.

## supplementary crystallographic information

### Comment

The pyridine-2,4-dicarboxylic acid (pdcH2) has important coordination functions to metals by either carboxylate bridges between metal centers, to form dimeric complexes or tridentate (O, N, O') chelation to metal ions. Some pydc complexes have been reported (Li *et al.*, 2007; Wang *et al.*, 2009; Aghabozorg *et al.*, 2011).

The title complex is isomorphous with the La^3+^ complex (Shen & Lush, 2011). The Ce^III^ is nine-coordinated in a distorted tricapped trigonal prismatic geometry formed by N and O atoms from a pydc ligand, two O atoms from two pydc ligands, two O atoms from one oxalate ligand and three O atoms from coordinated water molecules (shown as Fig. 1, Table 1). The mid-point of the oxalate anion is located on an inversion center. The oxalate and pyridinedicarboxylate anions bridge the Ce^3+^ cations to form the polymeric complex (Fig. 2).

The crystal structure contains O—H···O and weak C—H···O hydrogen bonds. The π-π stacking between two pyridine rings of (pydc) anion fragments with centroids distance of 3.558 (2) Å (1 - *x*, 1 - *y*, 1 - *z*) are observed. The uncoordinated water molecule shows half-occupation.

### Experimental

Ce(NO_3_)_3_.6H2O (0.1086 g, 0.25 mmole), pydridine-2,4-dicarboxylic acid (0.0418 g, 0.25 mmol) and 4,4'-dipyridine (0.0464 g, 0.25 mmol) were mixed in 10 ml of deionized water. After stirring for 30 min, the mixture was placed in a 23 ml Teflon-lined reactor which was heated under autogenous pressure to 418 K for 48 h and then allowed to cool to room temperature. The colorless transparent single crystals were obtained in 35.6% yield (based on Ce).

### Refinement

The site occupancy factor of the lattice water O10 was refined to 0.486 (15), and was set as 0.5 at the final cycles of refinement. Water H atoms were fixed in chemical sensible positions, thermal parameters were fixed as 0.08 Å^2^. Other H atoms were positioned geometrically with C—H = 0.93 Å (aromatic) and refined using a riding model with *U*_iso_(H) = 1.2*U*_eq_(C).

### Crystal data

**Table:**

[Ce_2_(C_7_H_3_NO_4_)_2_(C_2_O_4_)(H_2_O)_6_]·H_2_O	*Z* = 1
*M**_r_* = 824.58	*F*(000) = 398
Triclinic, *P*1	*D*_x_ = 2.398 Mg m^−^^3^
Hall symbol: -P 1	Mo *K*α radiation, λ = 0.71073 Å
*a* = 6.4160 (5) Å	Cell parameters from 4490 reflections
*b* = 6.6486 (6) Å	θ = 2.5–25.0°
*c* = 13.9920 (12) Å	µ = 4.04 mm^−^^1^
α = 89.917 (1)°	*T* = 294 K
β = 85.588 (1)°	Columnar, colorless
γ = 73.676 (1)°	0.30 × 0.10 × 0.10 mm
*V* = 570.98 (8) Å^3^	

### Data collection

**Table:**

Bruker SMART 1000 CCD area-detector diffractometer	2664 independent reflections
Radiation source: fine-focus sealed tube	2416 reflections with *I* > 2σ(*I*)
graphite	*R*_int_ = 0.030
Detector resolution: 9 pixels mm^-1^	θ_max_ = 27.8°, θ_min_ = 1.5°
φ and ω scans	*h* = −8→8
Absorption correction: multi-scan (*SADABS*; Bruker, 2001)	*k* = −8→8
*T*_min_ = 0.639, *T*_max_ = 0.937	*l* = −18→18
6072 measured reflections	

### Refinement

**Table:**

Refinement on *F*^2^	Secondary atom site location: difference Fourier map
Least-squares matrix: full	Hydrogen site location: inferred from neighbouring sites
*R*[*F*^2^ > 2σ(*F*^2^)] = 0.028	H-atom parameters constrained
*wR*(*F*^2^) = 0.081	*w* = 1/[σ^2^(*F*_o_^2^) + (0.0523*P*)^2^ + 0.4154*P*] where *P* = (*F*_o_^2^ + 2*F*_c_^2^)/3
*S* = 1.10	(Δ/σ)_max_ = 0.019
2664 reflections	Δρ_max_ = 2.75 e Å^−^^3^
177 parameters	Δρ_min_ = −2.70 e Å^−^^3^
0 restraints	Extinction correction: *SHELXTL* (Sheldrick, 2008), FC^*^=KFC[1+0.001XFC^2^Λ^3^/SIN(2Θ)]^-1/4^
Primary atom site location: structure-invariant direct methods	Extinction coefficient: 0.0071 (15)

### Special details

**Table:**

Geometry. Bond distances, angles *etc*. have been calculated using the rounded fractional coordinates. All su's are estimated from the variances of the (full) variance-covariance matrix. The cell e.s.d.'s are taken into account in the estimation of distances, angles and torsion angles
Refinement. Refinement on *F*^2^ for ALL reflections except those flagged by the user for potential systematic errors. Weighted *R*-factors *wR* and all goodnesses of fit *S* are based on *F*^2^, conventional *R*-factors *R* are based on *F*, with *F* set to zero for negative *F*^2^. The observed criterion of *F*^2^ > σ(*F*^2^) is used only for calculating -*R*-factor-obs *etc*. and is not relevant to the choice of reflections for refinement. *R*-factors based on *F*^2^ are statistically about twice as large as those based on *F*, and *R*-factors based on ALL data will be even larger.

### Fractional atomic coordinates and isotropic or equivalent isotropic displacement parameters (Å^2^)

**Table:**

	*x*	*y*	*z*	*U*_iso_*/*U*_eq_	Occ. (<1)
Ce	0.36484 (3)	0.33000 (3)	0.80343 (1)	0.0198 (1)	
O1	0.7620 (6)	0.3463 (6)	0.2798 (2)	0.0356 (11)	
O2	1.0901 (6)	0.2177 (7)	0.3373 (3)	0.0438 (13)	
O3	1.0817 (5)	0.2746 (6)	0.7009 (2)	0.0350 (10)	
O4	0.7595 (5)	0.3214 (7)	0.7822 (2)	0.0380 (13)	
O5	0.5054 (7)	0.2476 (5)	0.9655 (2)	0.0408 (13)	
O6	0.5773 (6)	0.3678 (5)	1.1047 (2)	0.0360 (10)	
O7	0.0036 (8)	0.5134 (8)	0.8992 (4)	0.0729 (14)	
O8	0.5654 (6)	−0.0533 (6)	0.7844 (3)	0.0419 (11)	
O9	0.1789 (8)	0.0870 (8)	0.8994 (4)	0.0729 (14)	
N1	0.5723 (5)	0.2600 (6)	0.6274 (2)	0.0208 (9)	
C1	0.7770 (7)	0.2748 (6)	0.6150 (3)	0.0218 (11)	
C2	0.5683 (7)	0.2406 (7)	0.4556 (3)	0.0248 (11)	
C3	0.7772 (7)	0.2622 (7)	0.4439 (3)	0.0227 (11)	
C4	0.8836 (7)	0.2747 (7)	0.5250 (3)	0.0251 (11)	
C5	0.4745 (7)	0.2392 (7)	0.5484 (3)	0.0256 (12)	
C6	0.8875 (8)	0.2745 (7)	0.3451 (3)	0.0275 (12)	
C7	0.8828 (7)	0.2921 (7)	0.7052 (3)	0.0246 (11)	
C8	0.5247 (7)	0.3884 (6)	1.0201 (3)	0.0237 (11)	
O10	−0.1094 (13)	−0.0862 (13)	0.9223 (7)	0.050 (3)	0.500
H2A	0.49300	0.22740	0.40290	0.039 (15)*	
H4A	1.02630	0.28290	0.51910	0.0300*	
H5A	0.33490	0.22280	0.55600	0.0300*	
H7A	−0.07800	0.47060	0.86320	0.0800*	
H7B	−0.02500	0.63680	0.88280	0.0800*	
H8A	0.53670	−0.16200	0.81820	0.0800*	
H8B	0.65420	−0.09500	0.73490	0.0800*	
H9A	0.24360	−0.06740	0.89420	0.0800*	
H9B	0.06500	0.09050	0.87300	0.0800*	
H10A	−0.21970	0.00790	0.90760	0.0800*	0.500
H10B	−0.12520	−0.12960	0.98560	0.0800*	0.500

### Atomic displacement parameters (Å^2^)

**Table:**

	*U*^11^	*U*^22^	*U*^33^	*U*^12^	*U*^13^	*U*^23^
Ce	0.0191 (2)	0.0294 (2)	0.0134 (1)	−0.0113 (1)	−0.0006 (1)	0.0005 (1)
O1	0.0380 (19)	0.044 (2)	0.0238 (16)	−0.0092 (16)	−0.0055 (14)	0.0092 (14)
O2	0.0270 (18)	0.064 (3)	0.0303 (19)	0.0011 (17)	0.0068 (15)	0.0078 (17)
O3	0.0199 (15)	0.061 (2)	0.0274 (17)	−0.0163 (15)	−0.0040 (13)	−0.0028 (15)
O4	0.0268 (17)	0.075 (3)	0.0185 (15)	−0.0250 (17)	−0.0004 (13)	−0.0012 (16)
O5	0.074 (3)	0.0266 (17)	0.0225 (16)	−0.0142 (17)	−0.0080 (17)	−0.0003 (13)
O6	0.058 (2)	0.0306 (17)	0.0222 (16)	−0.0141 (16)	−0.0143 (15)	0.0071 (13)
O7	0.055 (2)	0.061 (2)	0.088 (3)	−0.0018 (16)	0.0304 (19)	0.0251 (19)
O8	0.048 (2)	0.0333 (19)	0.041 (2)	−0.0115 (16)	0.0176 (17)	−0.0007 (15)
O9	0.055 (2)	0.061 (2)	0.088 (3)	−0.0018 (16)	0.0304 (19)	0.0251 (19)
N1	0.0174 (16)	0.0268 (17)	0.0193 (16)	−0.0077 (13)	−0.0030 (13)	0.0001 (13)
C1	0.0197 (19)	0.024 (2)	0.022 (2)	−0.0068 (16)	−0.0017 (15)	0.0017 (15)
C2	0.028 (2)	0.030 (2)	0.0179 (19)	−0.0099 (17)	−0.0049 (16)	−0.0011 (16)
C3	0.024 (2)	0.025 (2)	0.0162 (18)	−0.0024 (16)	−0.0005 (15)	0.0042 (15)
C4	0.0172 (19)	0.035 (2)	0.022 (2)	−0.0057 (17)	−0.0003 (16)	0.0045 (17)
C5	0.022 (2)	0.031 (2)	0.026 (2)	−0.0110 (17)	−0.0029 (16)	0.0014 (17)
C6	0.033 (2)	0.029 (2)	0.017 (2)	−0.0045 (18)	0.0037 (17)	−0.0004 (16)
C7	0.0196 (19)	0.033 (2)	0.025 (2)	−0.0129 (17)	−0.0041 (16)	0.0032 (17)
C8	0.028 (2)	0.023 (2)	0.0189 (19)	−0.0052 (16)	−0.0027 (16)	0.0009 (15)
O10	0.037 (4)	0.042 (4)	0.072 (6)	−0.010 (3)	−0.013 (4)	0.009 (4)

### Geometric parameters (Å, °)

**Table:**

Ce—N1	2.684 (3)	O8—H8B	0.8600
Ce—O1^i^	2.409 (4)	O9—H9B	0.8400
Ce—O3^ii^	2.505 (3)	O9—H9A	0.9900
Ce—O4	2.511 (3)	O10—H10A	0.8400
Ce—O5	2.508 (3)	O10—H10B	0.9400
Ce—O6^iii^	2.515 (3)	N1—C5	1.338 (5)
Ce—O7	2.568 (5)	N1—C1	1.343 (6)
Ce—O8	2.515 (4)	C1—C4	1.386 (6)
Ce—O9	2.582 (5)	C1—C7	1.498 (6)
O1—C6	1.268 (6)	C2—C3	1.385 (7)
O2—C6	1.244 (7)	C2—C5	1.390 (6)
O3—C7	1.245 (6)	C3—C6	1.517 (6)
O4—C7	1.268 (5)	C3—C4	1.383 (6)
O5—C8	1.248 (5)	C8—C8^iii^	1.543 (5)
O6—C8	1.253 (5)	C2—H2A	0.9300
O7—H7B	0.8300	C4—H4A	0.9300
O7—H7A	0.8600	C5—H5A	0.9300
O8—H8A	0.9200		
			
O4—Ce—O5	74.74 (12)	Ce—O5—C8	121.3 (3)
O4—Ce—O7	142.54 (15)	Ce^iii^—O6—C8	120.9 (3)
O4—Ce—O8	75.62 (14)	Ce—O7—H7B	105.00
O4—Ce—O9	130.01 (15)	H7A—O7—H7B	99.00
O4—Ce—N1	61.02 (10)	Ce—O7—H7A	96.00
O3^ii^—Ce—O4	137.32 (10)	Ce—O8—H8A	127.00
O1^i^—Ce—O4	94.76 (13)	H8A—O8—H8B	113.00
O4—Ce—O6^iii^	70.50 (13)	Ce—O8—H8B	119.00
O5—Ce—O7	84.31 (15)	Ce—O9—H9A	120.00
O5—Ce—O8	78.05 (12)	H9A—O9—H9B	96.00
O5—Ce—O9	67.22 (15)	Ce—O9—H9B	107.00
O5—Ce—N1	130.72 (12)	H10A—O10—H10B	112.00
O3^ii^—Ce—O5	141.57 (12)	Ce—N1—C5	123.6 (3)
O1^i^—Ce—O5	132.71 (11)	Ce—N1—C1	118.7 (2)
O5—Ce—O6^iii^	64.05 (10)	C1—N1—C5	117.1 (3)
O7—Ce—O8	130.23 (15)	N1—C1—C4	122.5 (4)
O7—Ce—O9	64.30 (16)	N1—C1—C7	115.4 (4)
O7—Ce—N1	144.66 (14)	C4—C1—C7	122.1 (4)
O3^ii^—Ce—O7	76.31 (14)	C3—C2—C5	118.1 (4)
O1^i^—Ce—O7	76.97 (15)	C2—C3—C6	121.4 (4)
O6^iii^—Ce—O7	72.47 (15)	C4—C3—C6	120.3 (4)
O8—Ce—O9	65.94 (15)	C2—C3—C4	118.3 (4)
O8—Ce—N1	71.24 (12)	C1—C4—C3	119.8 (4)
O3^ii^—Ce—O8	89.53 (13)	N1—C5—C2	124.1 (4)
O1^i^—Ce—O8	144.86 (12)	O1—C6—O2	127.1 (4)
O6^iii^—Ce—O8	134.19 (12)	O2—C6—C3	116.8 (4)
O9—Ce—N1	127.54 (14)	O1—C6—C3	116.0 (4)
O3^ii^—Ce—O9	74.47 (14)	O4—C7—C1	116.2 (4)
O1^i^—Ce—O9	134.92 (15)	O3—C7—C1	119.4 (4)
O6^iii^—Ce—O9	116.36 (14)	O3—C7—O4	124.4 (4)
O3^ii^—Ce—N1	76.35 (10)	O5—C8—O6	126.7 (4)
O1^i^—Ce—N1	74.55 (11)	O5—C8—C8^iii^	116.7 (4)
O6^iii^—Ce—N1	114.97 (11)	O6—C8—C8^iii^	116.6 (4)
O1^i^—Ce—O3^ii^	74.72 (12)	C5—C2—H2A	121.00
O3^ii^—Ce—O6^iii^	136.22 (12)	C3—C2—H2A	121.00
O1^i^—Ce—O6^iii^	68.95 (11)	C1—C4—H4A	120.00
Ce^i^—O1—C6	138.2 (3)	C3—C4—H4A	120.00
Ce^iv^—O3—C7	140.1 (3)	N1—C5—H5A	118.00
Ce—O4—C7	127.9 (3)	C2—C5—H5A	118.00
			
O5—Ce—O4—C7	158.0 (4)	N1—Ce—O1^i^—C6^i^	73.5 (5)
O7—Ce—O4—C7	−143.7 (4)	O4—Ce—O6^iii^—C8^iii^	−87.9 (4)
O8—Ce—O4—C7	76.7 (4)	O5—Ce—O6^iii^—C8^iii^	−5.9 (3)
O9—Ce—O4—C7	116.9 (4)	O7—Ce—O6^iii^—C8^iii^	86.3 (4)
N1—Ce—O4—C7	0.5 (4)	O8—Ce—O6^iii^—C8^iii^	−43.3 (4)
O3^ii^—Ce—O4—C7	3.6 (5)	O9—Ce—O6^iii^—C8^iii^	37.9 (4)
O1^i^—Ce—O4—C7	−68.9 (4)	N1—Ce—O6^iii^—C8^iii^	−130.9 (3)
O6^iii^—Ce—O4—C7	−134.6 (4)	Ce^i^—O1—C6—O2	−76.7 (7)
O4—Ce—O5—C8	80.8 (4)	Ce^i^—O1—C6—C3	101.7 (5)
O7—Ce—O5—C8	−67.9 (4)	Ce^iv^—O3—C7—O4	−12.4 (8)
O8—Ce—O5—C8	158.9 (4)	Ce^iv^—O3—C7—C1	168.6 (3)
O9—Ce—O5—C8	−132.4 (4)	Ce—O4—C7—O3	−174.5 (3)
N1—Ce—O5—C8	106.9 (4)	Ce—O4—C7—C1	4.5 (6)
O3^ii^—Ce—O5—C8	−127.4 (4)	Ce—O5—C8—O6	174.3 (4)
O1^i^—Ce—O5—C8	−1.5 (5)	Ce—O5—C8—C8^iii^	−4.7 (6)
O6^iii^—Ce—O5—C8	5.4 (4)	Ce^iii^—O6—C8—O5	175.0 (4)
O4—Ce—N1—C1	−6.1 (3)	Ce^iii^—O6—C8—C8^iii^	−6.0 (5)
O4—Ce—N1—C5	−176.6 (4)	Ce—N1—C1—C4	−169.4 (3)
O5—Ce—N1—C1	−35.2 (4)	Ce—N1—C1—C7	10.5 (5)
O5—Ce—N1—C5	154.3 (3)	C5—N1—C1—C4	1.7 (6)
O7—Ce—N1—C1	135.9 (3)	C5—N1—C1—C7	−178.4 (4)
O7—Ce—N1—C5	−34.6 (5)	Ce—N1—C5—C2	168.0 (3)
O8—Ce—N1—C1	−89.7 (3)	C1—N1—C5—C2	−2.7 (7)
O8—Ce—N1—C5	99.8 (4)	N1—C1—C4—C3	1.0 (6)
O9—Ce—N1—C1	−126.2 (3)	C7—C1—C4—C3	−178.8 (4)
O9—Ce—N1—C5	63.3 (4)	N1—C1—C7—O3	169.2 (4)
O3^ii^—Ce—N1—C1	176.1 (3)	N1—C1—C7—O4	−9.8 (6)
O3^ii^—Ce—N1—C5	5.6 (3)	C4—C1—C7—O3	−11.0 (6)
O1^i^—Ce—N1—C1	98.5 (3)	C4—C1—C7—O4	170.0 (4)
O1^i^—Ce—N1—C5	−72.0 (3)	C5—C2—C3—C4	2.0 (6)
O6^iii^—Ce—N1—C1	41.2 (3)	C5—C2—C3—C6	−177.4 (4)
O6^iii^—Ce—N1—C5	−129.3 (3)	C3—C2—C5—N1	0.9 (7)
O4—Ce—O3^ii^—C7^ii^	−172.6 (4)	C2—C3—C4—C1	−2.8 (7)
O5—Ce—O3^ii^—C7^ii^	49.5 (6)	C6—C3—C4—C1	176.5 (4)
O7—Ce—O3^ii^—C7^ii^	−12.4 (5)	C2—C3—C6—O1	27.1 (6)
O8—Ce—O3^ii^—C7^ii^	119.4 (5)	C2—C3—C6—O2	−154.4 (5)
O9—Ce—O3^ii^—C7^ii^	54.3 (5)	C4—C3—C6—O1	−152.3 (4)
N1—Ce—O3^ii^—C7^ii^	−169.8 (5)	C4—C3—C6—O2	26.3 (6)
O4—Ce—O1^i^—C6^i^	131.6 (5)	O5—C8—C8^iii^—O5^iii^	−180.0 (5)
O5—Ce—O1^i^—C6^i^	−154.8 (4)	O5—C8—C8^iii^—O6^iii^	−0.9 (6)
O7—Ce—O1^i^—C6^i^	−85.4 (5)	O6—C8—C8^iii^—O5^iii^	0.9 (6)
O8—Ce—O1^i^—C6^i^	60.0 (6)	O6—C8—C8^iii^—O6^iii^	180.0 (4)
O9—Ce—O1^i^—C6^i^	−54.6 (5)		

Symmetry codes: (i) −*x*+1, −*y*+1, −*z*+1; (ii) *x*−1, *y*, *z*; (iii) −*x*+1, −*y*+1, −*z*+2; (iv) *x*+1, *y*, *z*.

### Hydrogen-bond geometry (Å, °)

**Table:**

*D*—H···*A*	*D*—H	H···*A*	*D*···*A*	*D*—H···*A*
O7—H7A···O4^ii^	0.86	2.03	2.879 (6)	171.00
O7—H7B···O10^v^	0.83	1.84	2.569 (10)	146.00
O8—H8A···O6^vi^	0.92	2.00	2.910 (5)	170.00
O8—H8B···O2^vii^	0.86	1.84	2.655 (6)	159.00
O9—H9A···O6^vi^	0.99	2.01	2.987 (6)	169.00
O9—H9B···O10	0.84	1.93	2.440 (10)	118.00
O10—H10A···O5^ii^	0.84	2.12	2.844 (9)	143.00
O10—H10A···O8^ii^	0.84	2.39	2.913 (10)	121.00
O10—H10B···O9^viii^	0.94	1.63	2.501 (11)	153.00
C5—H5A···O3^ii^	0.93	2.46	3.131 (5)	129.

Symmetry codes: (ii) *x*−1, *y*, *z*; (v) *x*, *y*+1, *z*; (vi) −*x*+1, −*y*, −*z*+2; (vii) −*x*+2, −*y*, −*z*+1; (viii) −*x*, −*y*, −*z*+2.
